# Information Cascades and the Collapse of Cooperation

**DOI:** 10.1038/s41598-020-64800-z

**Published:** 2020-05-14

**Authors:** Guoli Yang, Attila Csikász-Nagy, William Waites, Gaoxi Xiao, Matteo Cavaliere

**Affiliations:** 1 Unit 66136 and College of System Engineering, National University of Defense Technology, Changsha, China; 2 Faculty of Information Technology and Bionics, Pázmány Péter Catholic University, Budapest, Hungary; 3 Randall Centre for Cell and Molecular Biophysics, King’s College, London, London, UK; 4 School of Informatics, University of Edinburgh, Edinburgh, Scotland UK; 5 School of Electrical and Electronic Engineering, Nanyang Technological University, Singapore, Singapore; 6 Department of Computing and Mathematics, Manchester Metropolitan University, Manchester, UK

**Keywords:** Social evolution, Complex networks

## Abstract

In various types of structured communities newcomers choose their interaction partners by selecting a role-model and copying their social networks. Participants in these networks may be cooperators who contribute to the prosperity of the community, or cheaters who do not and simply exploit the cooperators. For newcomers it is beneficial to interact with cooperators but detrimental to interact with cheaters. However, cheaters and cooperators usually cannot be identified unambiguously and newcomers’ decisions are often based on a combination of private and public information. We use evolutionary game theory and dynamical networks to demonstrate how the specificity and sensitivity of those decisions can dramatically affect the resilience of cooperation in the community. We show that promiscuous decisions (high sensitivity, low specificity) are advantageous for cooperation when the strength of competition is weak; however, if competition is strong then the best decisions for cooperation are risk-adverse (low sensitivity, high specificity). Opportune decisions based on private and public information can still support cooperation but suffer of the presence of information cascades that damage cooperation, especially in the case of strong competition. Our research sheds light on the way the interplay of specificity and sensitivity in individual decision-making affects the resilience of cooperation in dynamical structured communities.

## Introduction

Cooperation is widespread in the real world and can be observed at different scales of biological organization, ranging from genes to multi-cellular organisms and socio-technological systems^1–4^. However, cooperators pay a cost to benefit others. The extent to which cooperators can thrive within the system apparently contradicts the idea that only selfish behaviors are rewarded during competition between individuals. The resilience of cooperation has been approached in different domains^5^ and evolutionary game theory^6–8^ provides a framework for studying the evolution of cooperation among unrelated individuals.

The Prisoner’s Dilemma (PD) in particular has been widely employed for investigating the sustainability of cooperation. The prisoner’s dilemma stresses the key point of the conflict of interest between what is best for the individual and what is best for the group, and thus creates a social dilemma. To solve the dilemma, several mechanisms have been suggested to facilitate the spreading of cooperation such as direct reciprocity, indirect reciprocity, kin selection, group selection, and graph selection or spatial reciprocity, etc.^2^. Much work has been dedicated to the spreading of cooperation in structured populations and networks, where the promotion of cooperation is associated with the formation of cooperative clusters^9–12^.

In this paper we use evolutionary game theory to study a model of dynamical networks to understand how attachment choices cascade down the generations and lead, or not, to a collapse of cooperation. In this model, when a new node (newcomer) joins the network, it selects a role-model and imitates its strategy^13–15^. The newcomer also needs to decide its connections and, in particular, whether to establish connections with the social network of the chosen role-model, a mechanism often refereed as social inheritance^14–16^. A newcomer would ideally connect only to cooperative nodes and avoid detrimental connections to cheaters. The challenge for the newcomer, therefore, is to distinguish between cooperators and cheaters.

We model this challenge as a combination two kinds of information that we call *public* and *private*^17^. *Public* information is that which can be computed by observing the network and choices that previous newcomers have made.

We use the degree of a node for this public information, reasoning that if it is very popular, if more previous newcomers have connected to such node, then following the crowd might be a good idea. Note that the newcomer has no knowledge of *why* previous newcomers may have made this decision.

This is an important point: because newcomer nodes cannot know for certain whether nodes are cooperators or cheaters, and cannot know the reasons for previous newcomers connecting to a given node, they may erroneously select to connect to a cheater node.

This error increases the degree of that node and makes it more likely to be selected in the future. As this process continues, an information cascade can start - this same erroneous decision is copied on the basis of the *public* information again and again.

To temper this tendency to simply follow the crowd, with the potential for information cascades, newcomers also have access to *private* information. *Private* information gives a newcomer node a chance to act differently, to not follow the crowd. The *private* information is modelled simply as two Gaussian probability distributions, *ϕ*_*c*_ for cooperators and *ϕ*_*d*_ for cheaters^17^. The two distributions are then used by the newcomers to distinguish (using a decision-threshold) cooperators from cheaters. Different combinations of the distributions and of the decision-thresholds lead to decisions with different *specificity* and *sensitivity*. In this paper, we fix the two distributions, and change only the decision-thresholds. This allows us to study how different types of individual decisions (i.e., with different specificities and sensitivities) can affect the presence of cooperation. We investigate what happens when decisions are made based on private information alone as well as both private and public information^17^.

We demonstrate that different decisions based on private information, with different trade-offs of sensitivity and specificity, can have positive or negative consequences on long term cooperation and crucially depend on the strength of the competition between cooperators and cheaters (selection strength). Decisions beneficial for cooperation can become detrimental if the selection strength changes. Moreover we show that decisions based on a combination of private and public information create information cascades^17^ which generally damage cooperation, in particular when selection strength is strong.

## Computational Model

We consider a network of *N* nodes linked by a number of edges which varies over the course of the evolution of the system and where the individual success is linked to actual number and types of the neighbours^15,18,19^.

Each node is either a *cooperator* or *defector* (also referred as *cheater*), adopting one of the strategies of the *Prisoner’s Dilemma*^2,8,20^. A cooperator pays a cost *c* to distribute a benefit *b* to all of its neighbors. A defector pays no cost and distributes no benefit. We assume *b* > *c* > 0.

This can be represented by the following payoff matrix:

1 $$\begin{array}{cc} & \begin{array}{cc}C & D\end{array}\\ \begin{array}{c}C\\ D\end{array} & (\begin{array}{cc}b-c & -c\\ b & 0\end{array})\end{array}$$

The *payoff P*_*i*_ received by node *n*_*i*_ in the network is calculated as the sum of pair-wise interactions with its neighbours.

Specifically, if a cooperator has *m* cooperative neighbours and *n* defective neighbours, its payoff is $m(b-c)-nc$. However, a cheater in the same neighbourhood has payoff *mb*.

The *prosperity* of a population is defined by the average payoff of all nodes present in the network. We use an exponential function with a parameter *δ* to define the *fitness* of node *n*_*i*_, ${f}_{i}={\mathrm{(1}+\delta )}^{{P}_{i}}$ where *P*_*i*_ is the payoff of node *n*_*i*_.

**Figure 1:**
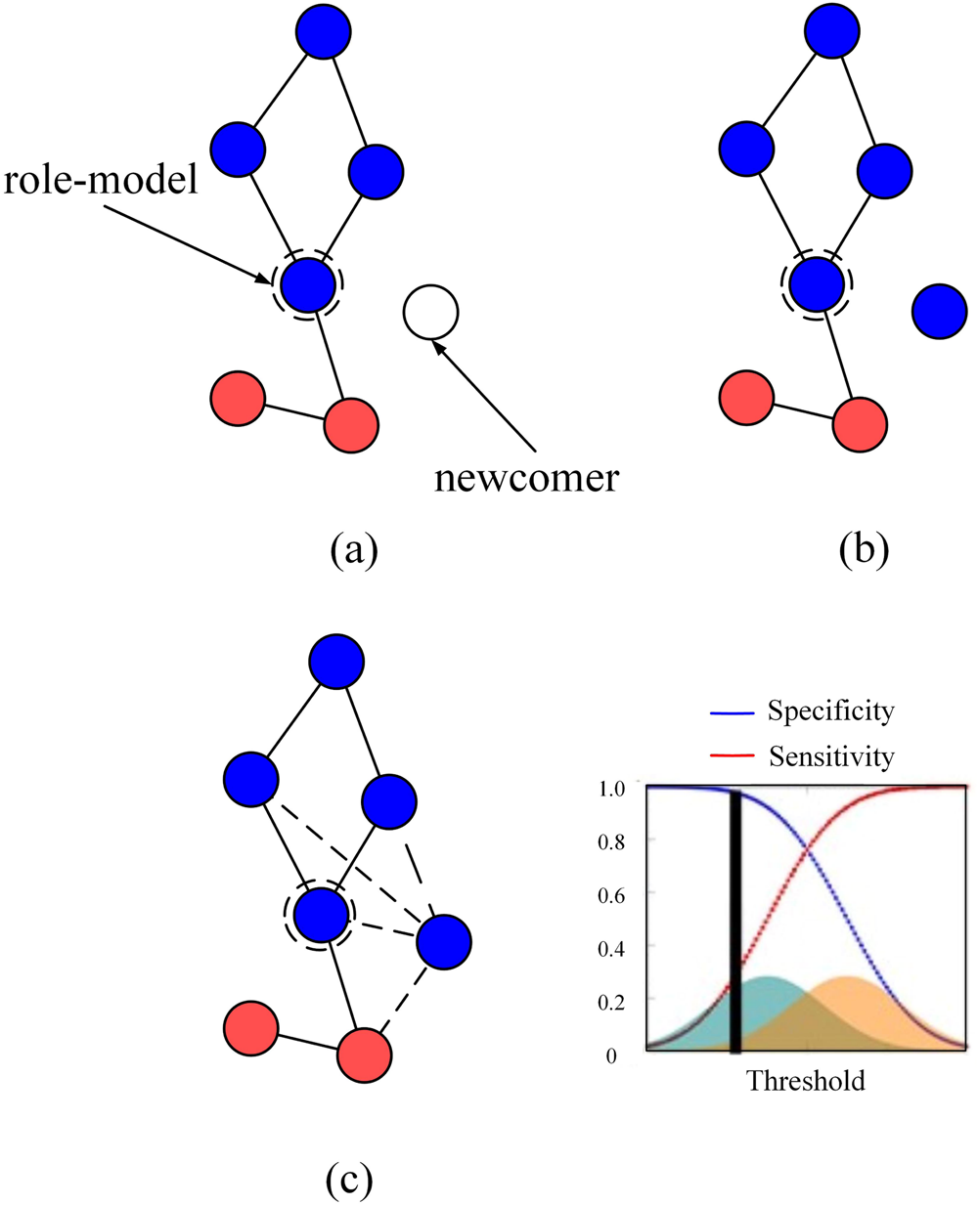
Network update. (**a**) A role-model is selected with probability proportional to its fitness. A newcomer (white node) is added to the network (**b**) The newcomer imitates the behaviour of the role-model with probability 1 − *μ*. (**c**) The newcomer establishes links (dashed) with the role-model and with each node of its social network with the aim to establish links only with the cooperators (blue nodes). The decision to establish a link with a node is based on the exclusive use of private (blue and orange distributions) or on a combination of private and public information which can be used by the newcomer to distinguish cooperative nodes.

Each *update step* of the network proceeds as follows (Fig. 1).

A *role-model* node is selected at random, with a probability proportional to its fitness (i.e., higher the fitness of a node, higher its chances to be selected as role-model). The parameter *δ* (*selection strength*) serves to determine the “strength” of the game between the nodes^21,22^. This means that when *δ* = 0, the selection probability is the same for all nodes, independently of their obtained payoff, while increasing *δ* makes it more likely that a node with a higher payoff is selected as role-model.

A *newcomer* node is added into the network and with a probability 1 − *μ* will behave exactly as the role-model (i.e., using the same strategy) while with a probability *μ* will behave differently adopting the alternative strategy. Here the parameter *μ* means the mutation rate, which usually is a small value (we use *μ* = 0.0001).

Once the newcomer is added, *edges are established* in two different ways, depending on decisions which can make use of either only (i) *private information* or (ii) *private and public information* (see Fig. 1) in order to identify the cooperators from cheaters.

### Decisions based on private information

Private information is modelled using fixed Gaussian distributions, ${\phi }_{c}={\mathscr{N}}({\mu }_{c},{\sigma }_{c}^{2})$ and ${\phi }_{d}={\mathscr{N}}({\mu }_{d},{\sigma }_{d}^{2})$, and use the variance of the standard Gaussian distribution, ${\sigma }_{c}^{2}={\sigma }_{d}^{2}=0.5$; we assume the distributions peaks are a distance of 1 apart, setting ${\mu }_{c}=-0.5$ and ${\mu }_{d}=0.5$. Individual decisions are modelled using a fixed real number $\tau$ (called the *decision-threshold*) chosen in the support of the private information distributions. Figure 1 shows the overlapping distributions ${\phi }_{c}$ (blue), ${\phi }_{d}$ (orange) and the vertical line denotes a possible decision-threshold between the two distributions.

Connections between the added newcomer and the rest of the network are established in the following way.

For each node *x* (either the chosen role-model or any of its neighbors) with whom a connection may be established, a sample, ${s}_{x}$ is drawn from the distribution ${\phi }_{c}$ or the other distribution ${\phi }_{d}$ accordingly as the node *x* is a cooperator or a defector. If ${s}_{x} < \tau$ for some threshold $\tau$, then a connection is established between the newcomer and the node *x* (the private information signals that *x* is cooperator); otherwise these two nodes would not be connected (the private information signals that *x* is a cheater).

In this paper we assume fixed private information distributions and analyzed different decisions by changing the decision-threshold $\tau$.

The use of the two overlapping distributions ${\phi }_{c}$, ${\phi }_{d}$ models the imperfect ability of the newcomer to observe and distinguish cooperative nodes from cheating ones.

### Decisions based on private & public information

We also consider the case in which connections are added based on a combination of both public and private information, which may give contradictory indications. We use a common and intuitive type of public information which is the *degree of a node*. In this way the public information provides information on the choices of previous newcomers: a node with many connections (high degree) has already attracted many connections from earlier newcomers and the chosen public information will indicate (perhaps erroneously) that the node is a cooperator and a connection to that node should be made.

Specifically, let *P* and *Q* be Boolean variables of whether or not the public and private signals indicate that a connection should be made, respectively. The private signal is just as specified above, and the public signal is “*does the target node x have a degree greater than the average degree of network*” (i.e., *k*_*x*_ > $\bar{k}$).

The private and public signals are then combined as follows:

1. If $P\wedge Q$, then connect
2. If $P\wedge \neg Q$, then connect with probability *p*
3. If $\neg P\wedge Q$, then connect with probability *q*
4. If $\neg P\wedge \neg Q$, then do not connect

where the parameters *p* and *q* control the weights of private information and public information when they conflict. There is a subtlety about the public signal. If the choice is made on the basis of and, the threshold comparison must be done as ≥. This is because otherwise it is never possible to recover from the scenario where there are no edges or connections. Similarly, if the choice is made as or, the comparison must be strictly greater than, otherwise if the network becomes fully connected it will always remain so.

### Node removal

In either cases (A) or (B), after the addition of a newcomer, following a Moran process^20^, a randomly selected node is removed from the network together with all of its connections so that number of nodes in the population is kept constant.

## Results

We study the proposed computational model using numerical simulations and computer visualizations, performed using custom created software tool.

Simulations start from a randomly connected network of *N* nodes having an average connectivity $k=4$. For each simulation run, we fix the decision-threshold $\tau$ to a value chosen uniformly from the range, [−2, 2]. All nodes initially adopt the same strategy and statistics are calculated by taking a single run of 10^8^ update steps as described in Computational Model Section.

We score long term cooperation (when there is no confusion, we simply say cooperation), prosperity and connectivity calculated as the sum of the fraction of cooperators, average payoff and average degree at each step, respectively, divided by the total number of steps considered in the simulation. We consider the normalized values (ranging between 0 and 1) of long term cooperation, prosperity and connectivity which are computed by dividing the respective values by the maximum possible.

We also record the numbers of *transitions*, i.e., switches between the two states of all cooperators and all cheaters. A trajectory of the system obtained in a simulation, with the typical switches between all cooperators and all cheaters is shown in Fig. 1 in Supplement.

Moreover, keeping in mind that newcomers would like to establish connections only with cooperative nodes, we record during the simulations the number of *false positive (FP)*, *false negative (FN)*, *true positive (TP)* and *true negative (TN)*.

If a newcomer decides to establish a connection with a cooperative node *x* then this constitutes a *TP*; if a newcomer decides to not establish a connection with a cooperative node, then it is a *FN*. If a newcomer decides to establish a connection with a cheater then it is a *FP*, while if a newcomer decides to not establish a connection with a cheater, then it is a *TN*. The corresponding *specificity* and *sensitivity* are defined as $$specificity=TN/$$$$(TN+FP)$$ and $$sensitivity=TP/(TP+FN)$$. We also refer to *1-specificity* as *promiscuity*. Distinct values of $$\tau $$ (decision-threshold) imply decisions with distinct sensitivity and specificity. Also note, that *specificity* measures how well cheaters are recognised by newcomers and *sensitivity* shows how properly cooperators are recognised.

### Which kinds of decisions help cooperation?

We first analyze the model where connections are established with decisions that depend exclusively on the available private information.

**Figure 2:**
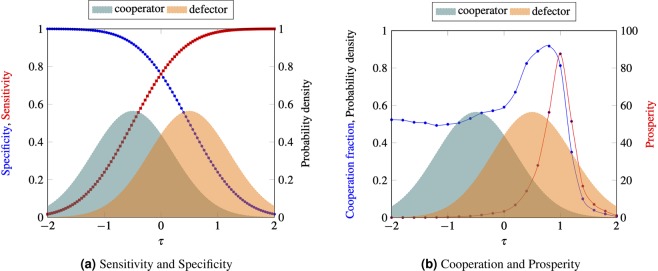
Decision-making based on private information and long-term cooperation. (**a**) The plot shows the sensitivity (red curve) and specificity (blue curve) of the decision associated to the chosen decision-threshold and the private information described by the two overlapping distributions. **(b**) The plot shows the amount of long-term cooperation (blue curve) and prosperity (red curve) obtained by varying the decision-threshold. Each chosen decision-threshold leads to a different decision which is associated a different specificity and sensitivity, leading to a different amount of long-term cooperation and prosperity. Results are obtained with a simulation of 10^8 ^ time steps using *δ* = 0.001 and *b*/*c* = 10/8.

We assume that all newcomers use an identical decision-threshold $\tau$. Varying the decision threshold $\tau$, one obtains decisions which differ for *sensitivity* and *specificity* (Fig. 2a); the sensitivity increases and the specificity decreases as the threshold increases. As one can see in Fig. 2b these differences affect the long term cooperation and prosperity present in the system, which clearly suggests that individual decisions based on an appropriate balance of specificity and sensitivity are important for the resilience of long term cooperation.

**Figure 3:**
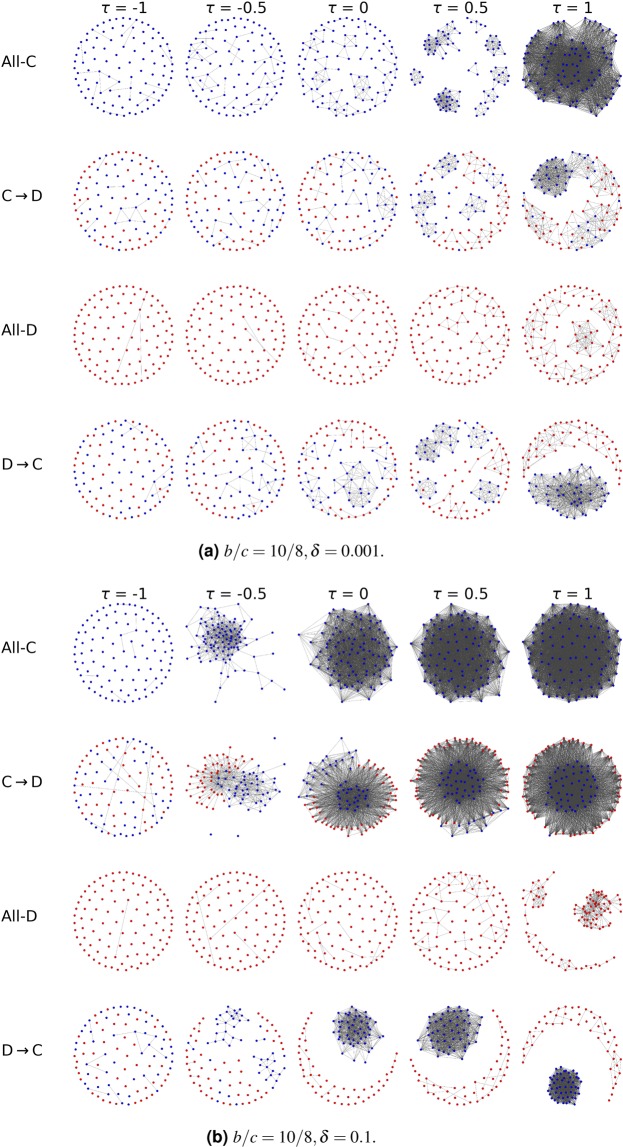
Typical networks for different decision-thresholds. We present the typical networks observed during a transition from of all cooperators to a network of all cheaters, and from all cheaters to all cooperators, for different decision-thresholds $\tau$. The typical stages of the invasions are: all-cooperators (All-C), cooperators-to-defectors (C → D), all-defectors (All-D) and defectors-to-cooperators (D → C), presented at $\tau =-1$, $\tau =-0.5$, $\tau =0$, $\tau =0.5$ and $\tau =1$. The simulations have been obtained at weak selection with $\delta =0.001$ (upper panel) and strong selection with $\delta =0.1$ (lower panel), and the benefit-to-cost ratio is high with *b*/*c* = 10/8.

To provide a better intuition on the effects of individual decisions ($\tau$) on long term cooperation we plot in Fig. 3 the network snapshots for different decision-thresholds and corresponding to the four key stages which we can find in a trajectory: a network of only cooperators, of only cheaters, the typical network in an invasion of cheaters (in a network of cooperators) and the typical network of an invasion of cooperators (in a network of cheaters). Layout of the networks is based on a physical model of springs^23^.

Figure 3 considers the case of large benefit-to-cost ratio (for the case of low benefit-to-cost ratio, see Fig. 2 in Supplement).

The collapse of a network of cooperators (All-C, C → D, All-D, Fig. 3) is generally facilitated by the introduction of a cheater into a well connected networks of cooperators. If a cheater “attaches” to a connected network of cooperators, such cheater would get a large fitness, attracting more cheating newcomers and ultimately leading to a full cheater invasion; we can see in Fig. 3 that this scenario is generally facilitated by increasing values of $\tau$ (increasing the promiscuity of the decisions). In that case, most connections to cooperators are established (leading to well connected networks) but those to cheaters are also rarely avoided (leading to frequent invasions of cheaters).

On the contrary, the recovery of cooperation from a network of a cheaters (All-D, D → C, All-C, Fig. 3) is fostered by a connected component of cooperators isolated in a sparse population of cheaters (with rare connections between the community of cooperators and that of cheaters). This scenario is facilitated by the lower values of $\tau$ (lower sensitivity of the decision). In that case, most detrimental connections to cheaters are correctly avoided and some connections to cooperators are also established.

However, as we can see in Fig. 3, the exact regime of decision-thresholds which avoid the formation of highly connected (and fragile) networks of cooperators, but still provide some sufficient connectivity between the cooperators, depend on the selection strength *δ*.

**Figure 4:**
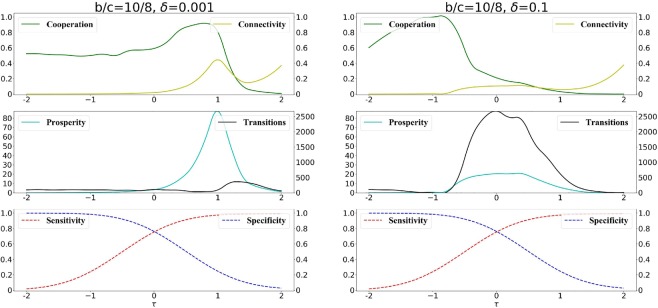
Cooperation, connectivity, prosperity and transitions as function of the decision threshold $\tau$. The beneficial or detrimental effects of decisions strongly depend on the selection strength. We use weak selection with $\delta =0.001$ (left panel) and strong selection with $\delta =0.1$ (right panel), respectively. The benefit-to-cost ratio is *b*/*c* = 10/8. Results are obtained using a long run of 10^8^ time steps.

We systematically explore the effects of the decision-threshold on cooperation in Fig. 4. In Fig. 4 we show how the amount of long term cooperation, the prosperity and the number of transitions change depending on the decision-threshold and the selection strength *δ*.

In general, cooperators can be successful if they avoid connections to cheaters and connect to other cooperators, so the best decisions for cooperation are at some intermediate level of specificity and sensitivity. However, analyzing Fig. 4 we can observe that the opportune balance of specificity and sensitivity depends crucially on the selection strength *δ*. Generally, decisions which are highly promiscuous (i.e., little specificity) tend to be negative for long term cooperation - however the opportune degree of promiscuity that maximizes cooperation depends on the strength of competition between cooperators and cheaters (selection strength).

The opportune balance of specificity and sensitivity is non trivial: detrimental decisions for cooperation can become beneficial if the selection strength changes. As we can observe in Fig. 4 when selection strength is weak, the maximum amount of long term cooperation is obtained when decisions are highly sensitive and promiscuous (Fig. 4, left panel). On the other hand, these types of decisions are detrimental for cooperation when the selection strength is strong (Fig. 4, right panel). In this case, the largest amount of cooperation is obtained when the individuals use decisions which are highly specific and with low sensitivity.

When benefit-to-cost ratio is generally smaller, the amount of long term cooperation is generally lower but also in that case the regimes of decisions which facilitate cooperation depends on the selection strength, and the decision-threshold $\tau$ after which cooperation collapses tend to smaller as selection strength increases (Fig. 3 in Supplement).

The intuition behind these results is the following one. For a given benefit-to-cost ratio *b*/*c* and a given selection strength *δ*, the connectivity and structure of the formed network is highly dependent on the threshold $\tau$ (as seen in Fig. 3). As we can see in Fig. 4 for both cases of weak and strong selections, there are intermediate $\tau$ s where long term cooperation is maximized. These are the $\tau$ s that facilitate the formation of networks where there is a good degree of connectivity (due to the sufficiently high promiscuity), a limited number of mixed connections between cooperators and cheaters (due to sufficiently high specificity), which are detrimental for cooperation. At the same time, these are the $\tau$ s that allow enough connections exclusively between cooperators (due to sufficiently high sensitivity) which are advantageous for cooperators. The optimal $\tau$ s for cooperation depend on the selection strength. Increasing $\tau$ leads to a decrease in the specificity and an increase in the sensitivity of the decisions. This means that, with the increase of $\tau$, the network tends generally to get more connected due an increase of the decision promiscuity (as we can observe in Fig. 3); we can observe a general increase in network connectivity and, in particular, due to the high promiscuity, an increase of the number of mixed connections between cooperative and cheating nodes (which are detrimental for cooperators and advantageous for cheaters). The detriment caused to cooperation by these types of mixed connections can be outbalanced by the connections which are exclusively between cooperative nodes (which are facilitate by high sensitivity) when the selection strength is weak (e.g., $\delta =0.001$, Fig. 4 left panel). However, for stronger selection (e.g., $\delta =0.1$, Fig. 4 right panel) the detrimental effects for cooperation of mixed connections (facilitated when $\tau$ is large) are larger: in fact, the chances that a cheater connected to cooperators is selected as role-model (and imitated by future newcomers) are larger as *δ* increases. Therefore, the decision-threshold $\tau$ corresponding to the best levels of cooperation decreases (higher specificity) with the increase of *δ* (this is true for large, Fig. 4, or small *b*/*c* Fig. 3 in Supplement).

Another interesting factor which is affected by the decision-threshold $\tau$ is the number of transitions between the two states of all cooperators and all cheaters. More transitions mean that the system is more unstable, with more frequent switches between the two states - cooperators invade networks of cheaters and cheaters invade networks of cooperators.

In Fig. 4 we can observe that the collapse of long term cooperation is correlated to an increase in the number of transitions - in fact, as $\tau$ increases, and the specificity decreases (promiscuity increases), there are more often connections between cooperators and cheaters which makes the network more unstable, with frequent invasions of cheaters which leads to an overall increase in the number of recorded transitions. Once the network is highly connected, however, at very high $\tau$ s, the cooperators cannot anymore recover (since it becomes harder to form up isolated cooperative communities), so the networks become more stable with rare invasions of cooperators, which corresponds to the observed low level of long term cooperation (Fig. 4). We can also observe that the prosperity (which is the long term overall payoff of the network) is maximized when there are sufficient cooperators and the network has some connectivity (only interactions produce some payoff) obtained when the specificity of the decisions is sufficiently low.

### Information cascades and the collapse of cooperation

As discussed in the Introduction, in many scenarios decisions are taken by considering a combination of public and private information; in this case a newcomer may make an erroneous decision to connect to a cheater when the private and public information conflict. We will investigate the influence of private & public information on the spreading of erroneous choices, and the consequences on long term cooperation.

As described above (computational model) we combine public and private information using the parameters *p* and *q* which control the decision-making of the newcomer when private and public information conflict. When *p* is larger than *q*, then the public information has a stronger weight in the decision to establish a connection, while when *q* is larger than *p*, the private information has a stronger weight in the decision.

The addition of public information can change the dynamics observed leading to the formation of *information cascades*^17^, that happen when an erroneous choice to connect (or not) to another node is made by the newcomer and such error can propagate to successive newcomers (a choice to connect is correct when the connection is established with a cooperator, while it is erroneous when established with a cheater).

We define two kinds of *information cascades*, *P*-cascades and *N*-cascades, depending on the different possibilities for the newcomer to establish (or not) its connections.

If the choice to connect or not is erroneous, the newcomer can be part of a cascade only if the private and public information conflict: if the private information correctly indicates that a connection *should be made* and a connection is nevertheless not made due to the public information, then the newcomer forms part of what we call a *P-cascade*. Similarly, if the private information correctly indicates that a connection *should not be made* and a connection is nevertheless made due to the public information, then the newcomer forms part of what we call an *N-cascade*.

The above situation is summarized in Table 1.

**Table 1:** Truth table for determining when a node forms part of a *P*-cascade or *N*-cascade.

correct	pub	priv	choice	cascade
C	C	C	C	0
C	NC	NC	NC	0
C	C	NC	NC	0
C	NC	C	C	0
C	C	NC	C	0
C	NC	C	NC	P
NC	C	C	C	0
NC	NC	NC	NC	0
NC	C	NC	NC	0
NC	NC	C	C	0
NC	NC	C	NC	0
NC	C	NC	C	N

The first column indicates whether the choice to connect (C) or not (NC) is correct. The second and third columns show the indication of the public and private information. The fourth column is the actual choice that is made. The final column identifies what sort of cascade this combination of inputs represents, either 0 for not a cascade, or *P*-cascade or *N*-cascade.

**Figure 5:**
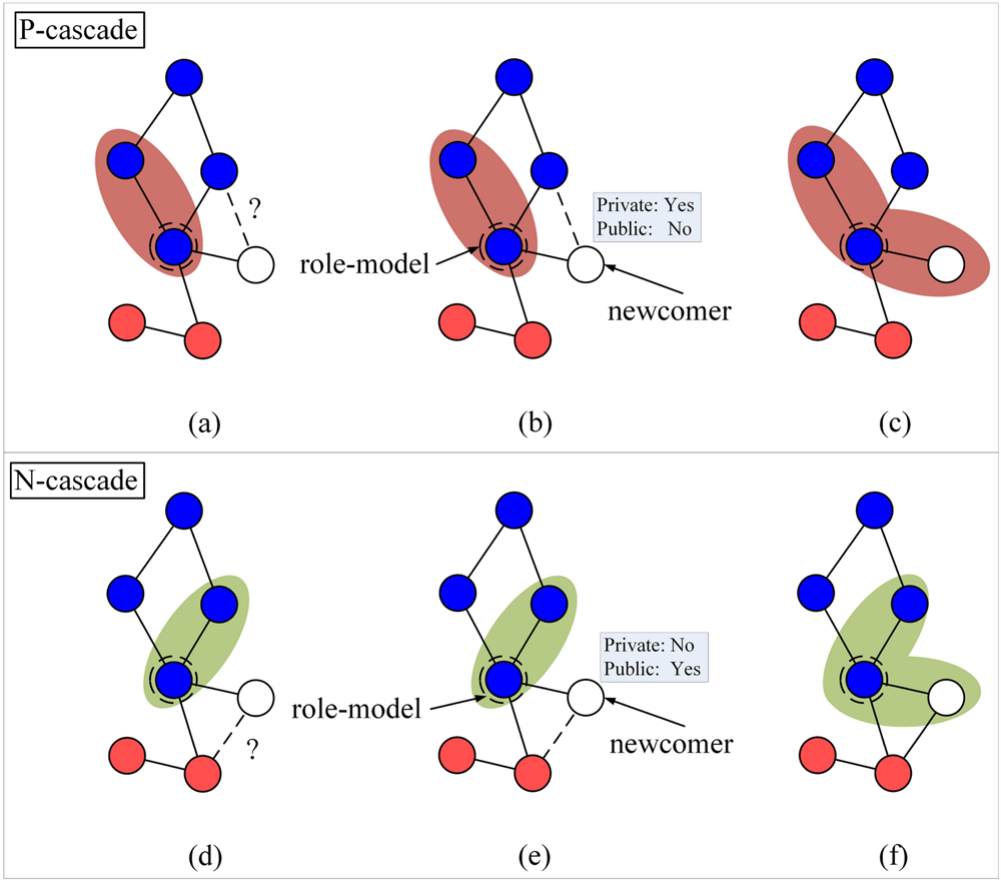
Growth of Information Cascades in the presence of Public Information. P-cascade: (**a**) A newcomer (next to the box) is added to the network and establishes a connection with the chosen role-model (without loss of generality we assume the role-model is part of a P-cascade - shaded light red area). (**b**) The newcomer decides on the other connections. **(c)** If the connection with a cooperator is missed as consequence of public information, then the newcomer becomes part of a P-cascade. N-cascade: (**d)** A newcomer is added to the network and establishes a connection with the chosen role-model (we assume the role-model is part of an N-cascade - light green shaded area). (**e**) The newcomer decides on the other connections. (**f**) If the connection with a cheater is established as consequence of the public information, then the newcomer becomes part of an N-cascade. In both types of cascades, the newcomers becomes part of the same cascade of its role-model if the type of their cascades is equal.

When a newcomer is determined as part of a cascade, its chosen role-model is then considered. If the type of the cascade, *P*-cascade or *N*-cascade is the same as that of the role-model, then the newcomer becomes part of the same cascade. If the type differs (or the role-model is not part of any cascade) then the newcomer becomes part of a new cascade (Fig. 5).

The structure of the network is affected by the type of information (private or public) which is predominant in the decisions and which can determine the amount of long term cooperation (see the typical trajectories of the system, with more or less frequent switches, as function of the predominant type of information, Fig. 4 in the Supplement).

**Figure 6:**
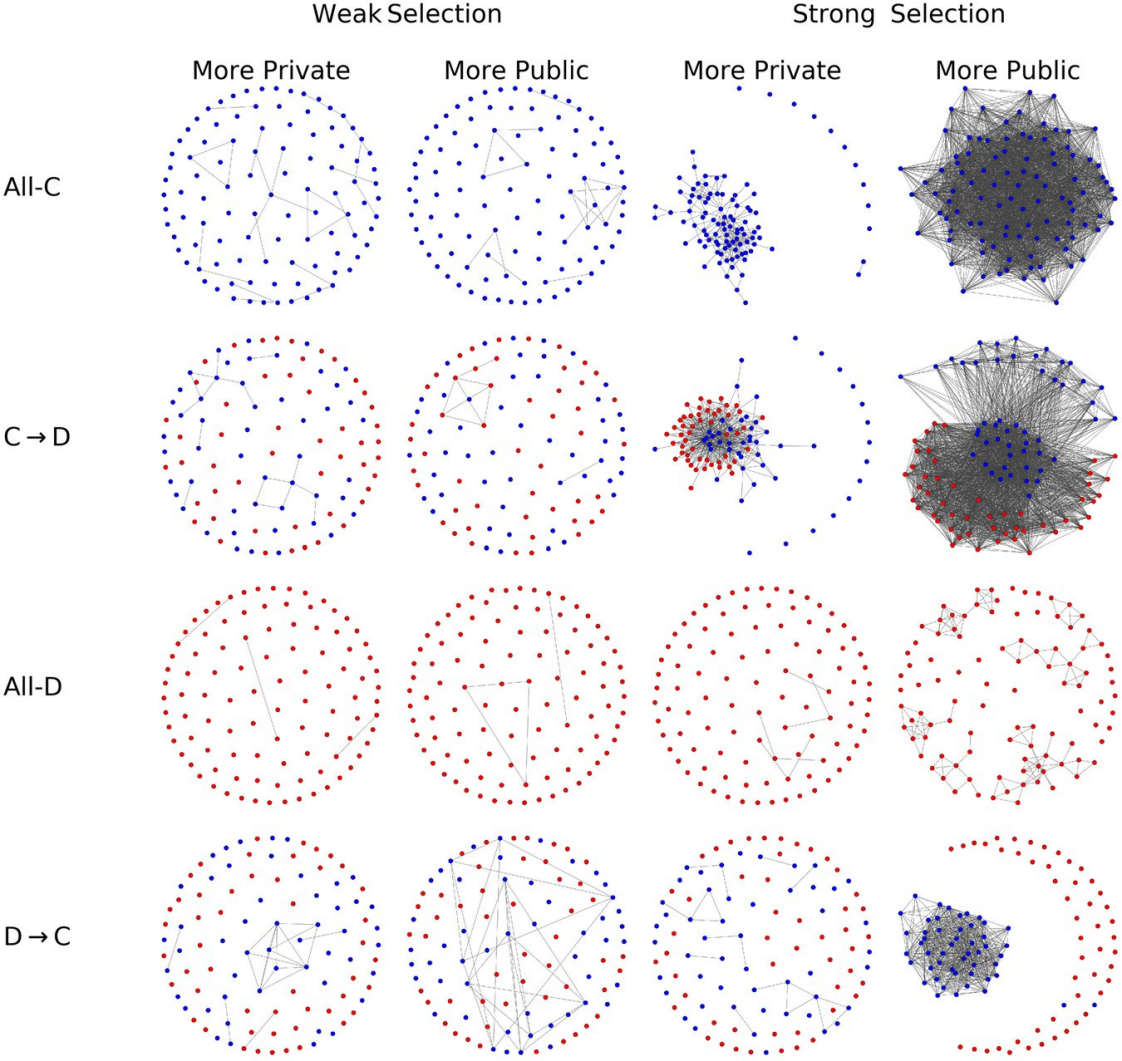
Networks structures when private or public information is predominant in the decisions. The snapshots represent the typical networks composed by all cooperators, all cheaters and the transients between these two states. We consider strong selection ($\delta =0.1$) and weak selection ($\delta -0.001$). The benefit-to-cost ratio is *b*/*c* = 10/8. More public information is obtained using $p=\mathrm{0.75,}\,q=0.25$. More private information is obtained with ($p=0.25$ and $q=0.75$). Decision-threshold *τ* is fixed to −1.

In Fig. 6 we plot the typical networks that can be observed in a trajectory of the system, corresponding to only cooperators, only cheaters and the transients between these two stages. We fix the decision-threshold and analyze the network changes for the cases in which private information is predominant in the decisions and the cases in which public information is predominant, in the scenarios of weak and strong selection.

Generally, we can see from Fig. 6 that connections are more likely to be established when more public information is considered in the decisions. This high connectivity caused by the presence of public information is particularly evident in the case of strong selection (rightmost panels in Fig. 6). For a smaller *b*/*c* ratio, we observe a similar scenario where strong selection and an increasing weight of public information foster the connectivity of networks (Fig. 5 in Supplement).

**Figure 7:**
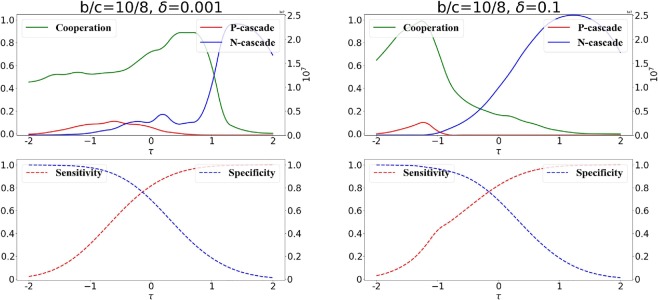
Information cascades with prevalent private information. We consider the scenario in which decisions are taken mostly (but not completely) based on private information ($p=\mathrm{0.25,}\,q=0.75$). We plot the long-term cooperation, total size of *P*-cascades and *N*-cascades. The benefit-to-cost ratio is *b*/*c* = 10/8, weak selection is $\delta =0.001$ (left panel) and strong selection is $\delta =0.1$ (right panel) are considered respectively.

We analyze in more details the consequences of public information on long term cooperation in Fig. 7. As one might intuitively expect, when private information is prevalent in the decisions, the curves are similar to the case where exclusively private information is present (Fig. 4).

However even a limited presence of public information already highlights a problem for long term cooperation: some mixed connections between cooperative and cheating nodes (which are detrimental for cooperators and advantageous for cheaters) are established due to the presence of public information. Therefore the way to balance these connections caused by public information is to increase the specificity (and consequently decreases the sensitivity) of the decisions: in fact, by comparing Figs. 4 and 7 we can observe that the maximal amount of long term cooperation is obtained at a lower *τ* when public information is also considered for the decisions.

We can also observe in Fig. 7 that the collapse of cooperation is correlated with an increase of the size of $N-cascades$, and the increase of the size of N-cascades start at lower *τ* for stronger selection strength *δ* (Fig. 7, right panel). This is essentially caused by the fact that once an *N*-cascade starts (an erroneous connection is established with a cheating role-model), then the cascade will grow as long as the same role-model is chosen by more newcomers, and this will happen with higher chances for stronger selection strength. The case for lower *b*/*c* ratio is presented in the Supplement (Fig. 6 in Supplement).

**Figure 8:**
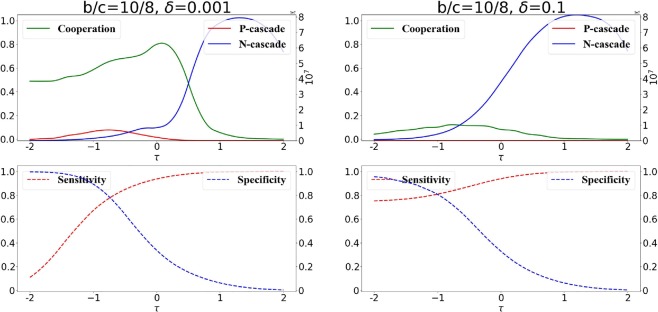
Information cascades with prevalent public information. We consider the scenario in which decisions are taken mostly (but not completely) based on public information ($p=\mathrm{0.75,}\,q=0.25$). We plot the long-term cooperation, total size of *P*-cascades and *N*-cascades. The benefit-to-cost ratio is *b*/*c* = 10/8, weak selection is $\delta =0.001$ (left panel) and strong selection is $\delta =0.1$ (right panel) are considered respectively.

In Fig. 8 we focus on the scenario in which decisions are taken based on public and private information with the weight of public information prevalent over the one of private information (the case of a lower benefit-to-cost ratio can be found in the Supplement Fig. 6). In general, comparing with Fig. 7, where little public information is used, we can see more dramatic effects. In the case of decisions where public information has a strong weight, long term cooperation decreases, particularly in the case of strong selection (Fig. 8, right panel).

**Figure 9:**
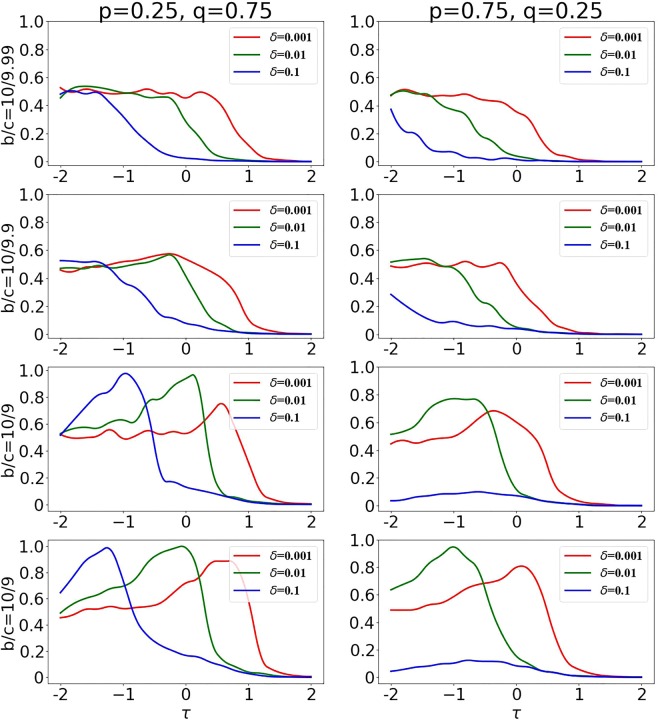
Public information facilitates the collapse of cooperation. We consider the scenario where mostly private information is used in the decision (left panels, with $p=\mathrm{0.25,}\,q=0.75$), and the scenario where mostly public information is used in the decisions ($p=\mathrm{0.75,}\,q=0.25$ (right panels)). We plot the the average amount of cooperators obtained in a long run for different *τ*s. We use $b/c=\mathrm{10/9.99}$ (first row), *b*/*c* = 10/9.9 (second row), $b/c=\mathrm{10/9}$ (third row) and $b/c\mathrm{=10/8}$ (bottom row). The selection strength is $\delta =0.001$, $\delta =0.01$ and $\delta =0.1$.

A stronger role of public information coupled to strong selection strength lead to an increase in the number of connections to cooperators at lower *τ*s; this increases the sensitivity of the decisions for low *τ*s (Fig. 8) because, in that regime, well connected nodes are mostly cooperators (due to the low number of false positives). This tendency of cooperators to be well connected is reinforced by the presence of public information: more connected nodes tend to attract new connections. The increase in sensitivity for lower *τ*s when public information has a strong weight makes difficult to find a balance of low sensitivity and high specificity which advantages cooperators (possible when mostly private information is used in the decisions, Fig. 7). In fact, as *τ*s increases, we observe a further increase of sensitivity (as the number of false negative decreases) and a sharp decrease in specificity (as the number of false positive increases - cheaters become highly connected and attract connections due to the presence public information). This is facilitated even more by the presence of information cascades whose size rapidly increases as *τ* increases and that lead to frequent formation of mixed connections between cooperators and cheaters (detrimental to cooperators). This is particularly evident when selection strength is strong (Fig. 8, right panel); when selection strength is weak, the detrimental effects of public information are less dramatic, as the chances of role-model selection are less connected to actual node fitness and this minimizes the growing of information cascades when the sensitivity of the decision is sufficiently large (Fig. 9, left panel).

Figure 9 summarizes the effects of public information - for different *b*/*c* and selection strengths: increasing the weight of public information in the decision is detrimental for long-term cooperation, with a more dramatic effect when the selection strength is large.

## Discussion

We analyzed a system where role-models are chosen by newcomers which imitate their strategy (cooperator or cheater) and their social connections inspired by the idea of social inheritance^15,16^. In general, for any newcomer (independently of its strategy) it is beneficial to connect to cooperators but detrimental to connect to cheaters. On the other hand, it is not possible to unambiguously identify cooperators from cheaters and the decisions to establish a connection become crucial for the resilience of cooperation. In this paper we evaluate the effects of different decisions expressed in terms of specificity and sensitivity. We show that the kinds of decisions (i.e., opportune balance of specificity and sensitivity) which maximize long term cooperation crucially depend on the degree of competition between cooperators and cheaters (selection strength). There is not really an unique kind of decisions that maximize cooperation: decisions which are beneficial for cooperation become detrimental if the selection strength changes.

When decisions are based exclusively on private information, we identify two major classes of decisions advantageous for cooperation: promiscuous decisions (high sensitivity, low specificity) where the priority is to properly connect to other cooperators rather than to avoid cheaters, and more risk-adverse decisions (high specificity, low sensitivity), where the priority is to avoid cheaters rather than connect to cooperators. Promiscuous decisions help cooperation when the selection strength is weak, while they are detrimental in the case of strong selection. In the latter case, the best decisions for cooperation are the risk-adverse ones (Fig. 4). Intuitively, when competition is strong (large selection strength) an erroneous mixed connection between a cooperator and a cheater can have large negative consequences on cooperation - in that case more risk adverse decisions, which carefully avoid cheaters, can benefit cooperation.

The addition of public information makes this effect more dramatic (Fig. 8): when the public information and private information conflict, and the decision to connect is based on a combination of the two, then an initial erroneous connection between a cooperator and a cheater can lead to the formation of information cascades^17^, e.g., a series of erroneous connections. Such information cascade generally facilitate connections of (cooperative and cheating) newcomers to cooperative nodes. This is advantageous for cooperative newcomers but even more for cheaters: it increases the sensitivity of the decisions, making difficult to find the opportune balance of sensitivity and specificity advantageous for cooperation. Though the negative effects on cooperation of public information are more evident when selection strength is strong, they can be observed independently of the selection strength and of the benefits and costs of cooperation (Fig. 9).

Overall, our results demonstrate the importance of decision-making in the resilience of cooperation in structured populations: whether it is better for cooperators to focus on avoiding cheaters, or to search for other cooperators, really depends on how strong is the competition. Though our work has been focusing on a specific dynamical network model, our approach is general and serves as a first step to shed light on the dynamics of structured systems where the time-scale of decision-making and strategies dynamics may overlap such as biological^24–26^ and social systems^27^. As the acquisition of information (public or private) is generally costly, it would be interesting to extend the considered model with an additional cost for the individuals related to their ability to acquire information (and to process them^28^). Furthermore the observed strategy separation (observed in Figs. 6 and 3) underlying our results has been observed in other models, e.g.^3,29^, which may suggest the possible generality of our results.

More generally, we believe that the proposed idea of coupling specificity/sensitivity trade-off of decision-making with evolutionary game theory in dynamical networks can be applied to other types of models, in a variety of scenarios where has already been highlighted the role of individual decisions in preserving cooperation^25,26,30–32^.

## Supplementary information

Supplementary information.
