# Supplementary material for: Information Cascades and the Collapse of Cooperation
Source: Sci Rep. 2020 May 14;10:8004. doi: 10.1038/s41598-020-64800-z (PMC7224182; doi:10.1038/s41598-020-64800-z)
Supplement: Supplementary file 1 — Supplementary information. [file 41598_2020_64800_MOESM1_ESM.pdf]

Information Cascades and the Collapse of Cooperation
Supplementary Material

Guoli Yang, Attila Csikasz-Nagy, William Waites, Gaoxi Xiao, Matteo Cavaliere

1 Decision-making based only on private information

1.1 Typical trajectories

When decision-making is based on private information, for a given benefit-to-cost ratio b/c and a given selection strength δ, the amount of long term cooperation and connectivity is dependent on the threshold τ, see Figure 1. With the increase of the decision-threshold the network gets more connected due to the decrease of specificity, but can be invaded by cheaters more easily. As discussed in the main text, long term cooperation is maximized at some intermediate τs, with an opportune level of specificity and sensitivity, which facilitate the spreading of cooperation meanwhile inhibit the growth of defection. In Figure 1 we plot the typical trajectories of the network, with switches between configurations of all cooperators and of all cheaters.

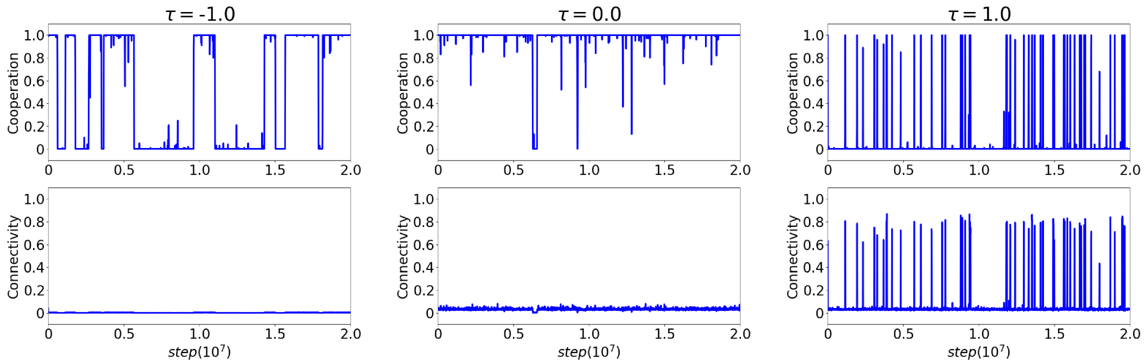

Figure 1: Typical trajectories of the network. We plot the amount of cooperation (normalized number of cooperators present in the network) and connectivity (average connectivity of the network) for $\tau = -1$, $\tau = 0$ and $\tau = 1$. The simulations have been obtained using $\delta = 0.01$ and $b/c = 10/9$.

1.2 Low benefit-to-cost ratio: typical networks

Figure 2 illustrates the typical networks for low benefit-to-cost ratio; as discussed in the main text, the collapse of cooperation is led by the introduction of cheaters connected to many cooperators which can happen more frequently at high values of τ and for strong selection.

1.3 Low benefit-to-cost ratio: cooperation

In Figure 3 we present the amount of long term cooperation, connectivity, prosperity and transitions for low benefit-to-cost ratio. As in the case of higher benefit-to-cost-ratio (presented in the main text) we can see that the collapse of cooperation starts at a lower τ as the selection becomes stronger.

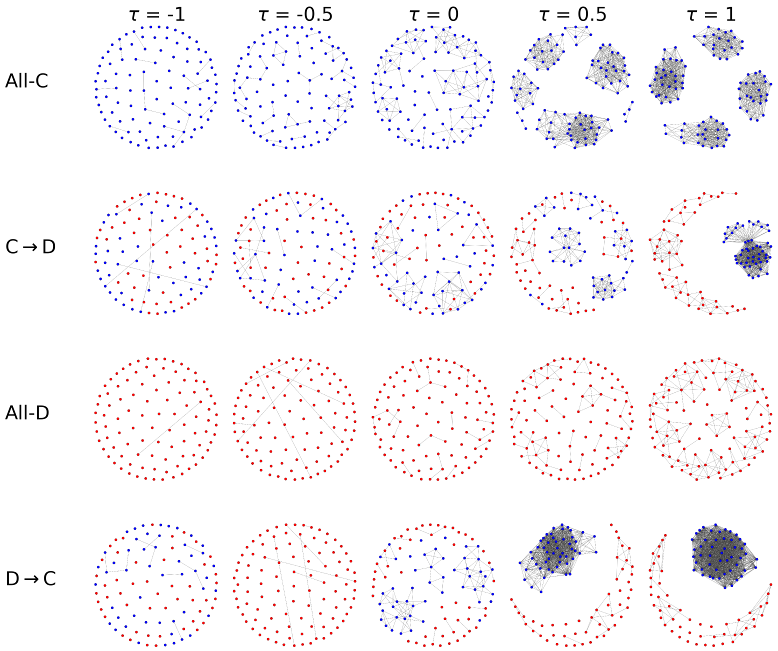

(a) Weak selection

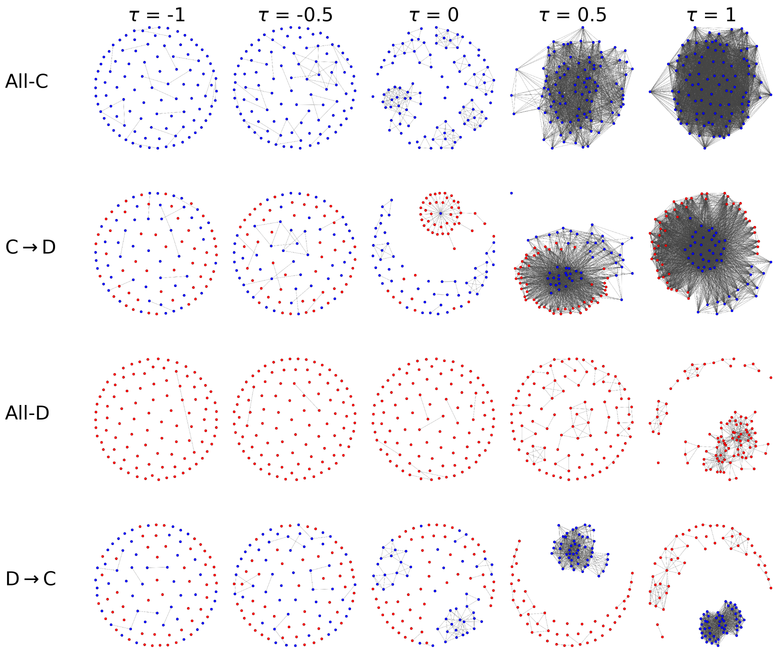

(b) Strong selection

Figure 2: Typical networks for different τs for weak and strong selection with $b/c = 10/9.9$. We show the typical networks with all-cooperators (top row), cooperators-to-defectors (second row), all-defectors (third row) and defectors-to-cooperators (bottom row) for $\tau = -1$, $\tau = -0.5$, $\tau = 0$, $\tau = 0.5$ and $\tau = 1$. The simulations have been obtained at $\delta = 0.001$ (left panel) and $\delta = 0.1$ (right panel).

2 Decision-making based on private and public information

As discussed in the main text, long term cooperation is strongly affected (and possibly damaged) by decisions based on the combination of private and public information. In Figure 3 we show the amount of long term

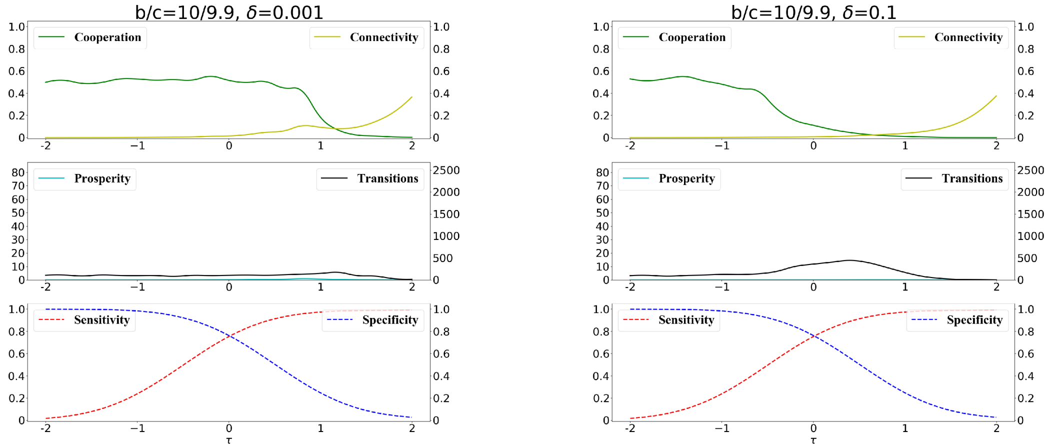

Figure 3: Long term cooperation, connectivity, prosperity and transitions as function of τ. The benefit-to-cost ratio is $b/c = 10/9.9$, weak selection $\delta = 0.001$ (left panel), strong selection $\delta = 0.1$ (right panel). Results are obtained by running simulations of 10^8 steps.

cooperation, connectivity, prosperity and number of transitions as function of the decision-threshold τs.

2.1 Long term cooperation and connectivity

When decisions are based on private and public information the amount of long term cooperation is highly dependent on the parameters p and q (which regulates the weight of the public information, as discussed in the main text) as well as by the decision-threshold τ (Figure 4).

With the increase of p and the decrease of q the public information has a stronger weight in the decision. As discussed in the main text, this can lead to erroneous connections to cheaters (and information cascades) which damage long term cooperation.

2.2 Typical networks

In Figure 5 we illustrate the typical networks for the case when private information has a stronger weight in the decision and the case in which public information has a stronger weight in the decision.

2.3 Cooperation and information cascades

In Figure 6 we present the amount of long term cooperation, connectivity, prosperity and transitions for low benefit-to-cost-ratio.

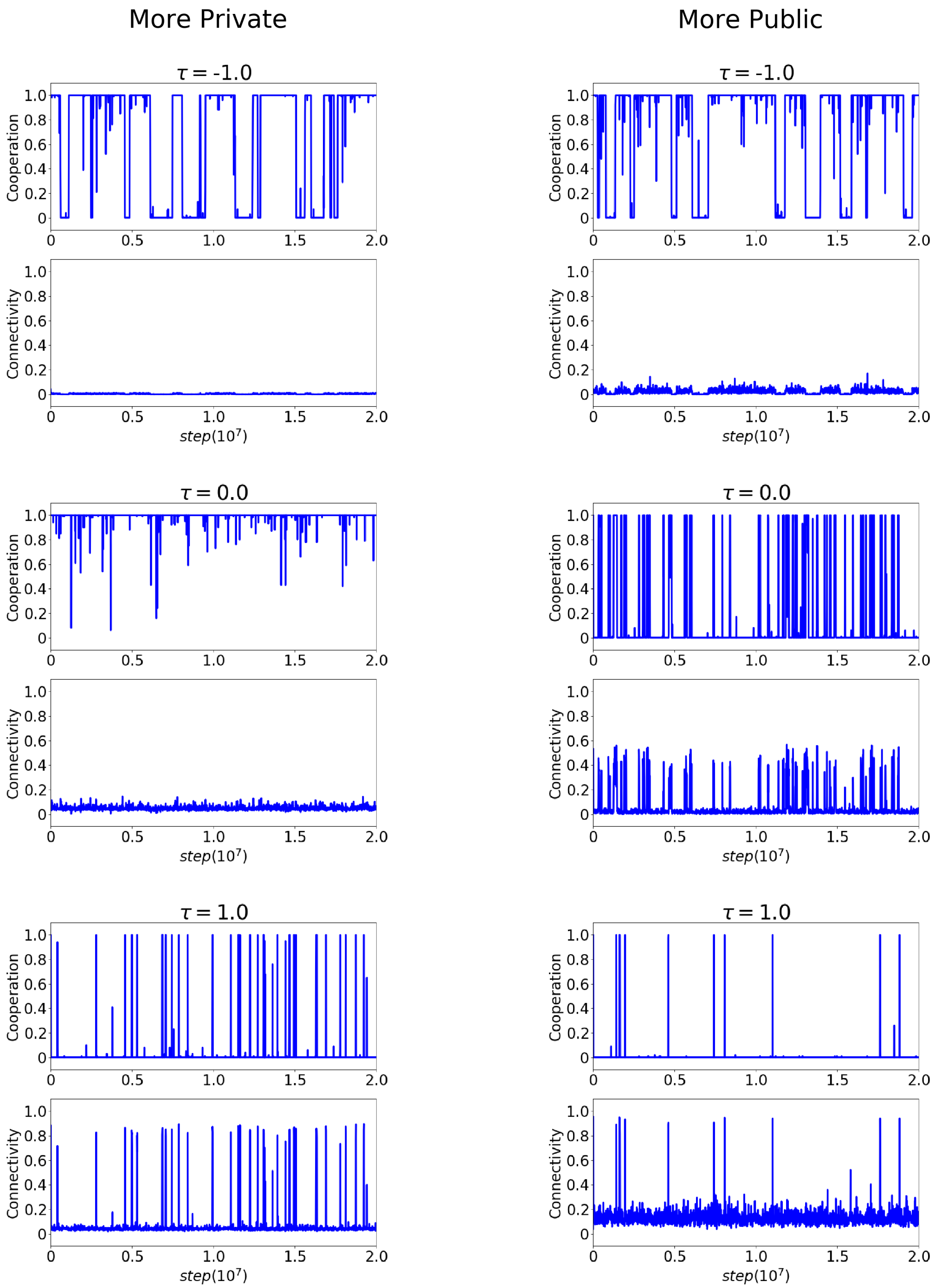

Figure 4: Typical trajectories of the system, showing cooperation (number of cooperators in the network) and connectivity obtained at $\tau = -1$, $\tau = 0$ and $\tau = 1$. We can see that a stronger weight for public information (right panel, $p = 0.75$ and $q = 0.25$) damages cooperation (the case for a stronger weight for private information is on left panel, $p = 0.25$ and $q = 0.75$). The simulations have been obtained for $\delta = 0.01$ and $b/c = 10/9$.

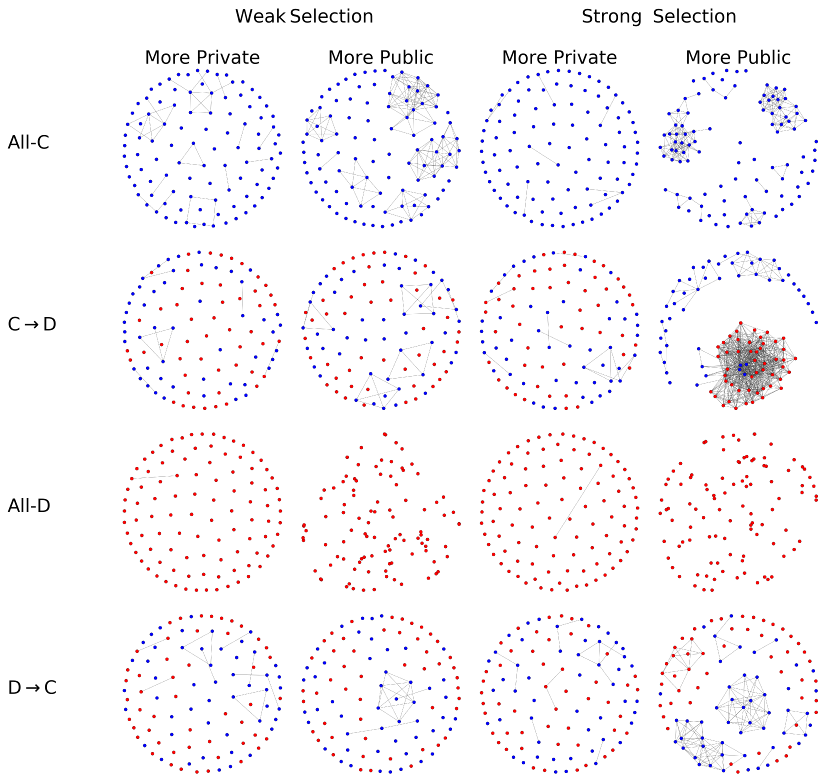

Figure 5: Typical networks obtained for $\tau = -1$, $b/c = 10/9.9$ and weak/strong selection. We show the typical networks with all-cooperators (top row), cooperators-to-defectors (second row), all-defectors (third row) and defectors-to-cooperators (bottom row). The decisions where private information is stronger ($p = 0.25$ and $q = 0.75$) and the decisions where public information is stronger ($p = 0.75$ and $q = 0.25$) are compared for weak selection ($\delta = 0.001$) and strong selection ($\delta = 0.1$).

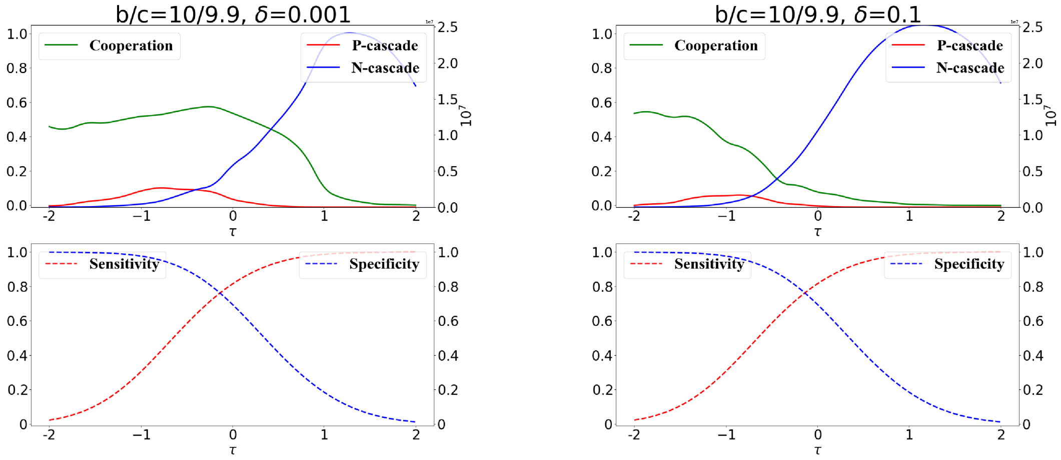

(a) More private information

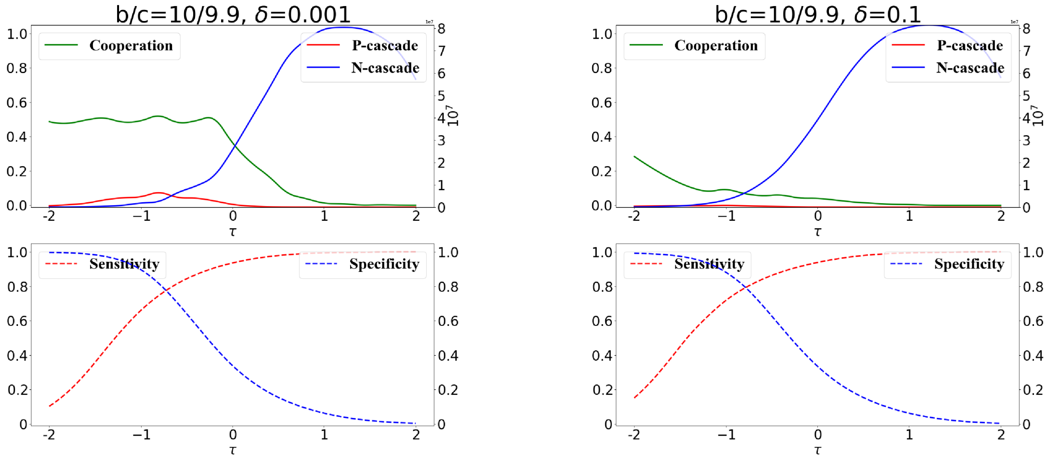

(b) More public information

Figure 6: Long term cooperation, connectivity, prosperity and transitions as function of τs. The case of decisions with stronger public information is presented in the lower panel ($p = 0.75$ and $q = 0.25$). The case for stronger private information is presented in the upper panel ($p = 0.25$ and $q = 0.75$). The benefit-to-cost ratio is $b/c = 10/9.9$ and we consider weak selection ($\delta = 0.001$, left panel) and strong selection ($\delta = 0.1$, right panel). Results are obtained with simulations of 10^8 steps.
